# Orchio-Septopexy: A new technique to cover and fix detorsed testis undergoing fasciotomy of tunica albuginea

**DOI:** 10.1590/S1677-5538.IBJU.2022.0128

**Published:** 2022-03-16

**Authors:** Mohammed Elifranji, Tariq Abbas, Bruno Leslie, Santiago Vallasciani, Abderrahman El Kadhi, João Luiz Pippi-Salle

**Affiliations:** 1 Department of Surgery Division of Pediatric Urology Sidra Medicine Doha Qatar Department of Surgery, Division of Pediatric Urology, Sidra Medicine, Doha, Qatar

**Keywords:** Reproductive Techniques, Fasciotomy, Penile Induration

## Abstract

**Purpose:**

Compartment Syndrome (CS) has been recognized as a potential factor that worsens testicular viability after detorsion, especially in borderline cases of prolonged ischemia. Fasciotomy of the testicular tunica albuginea to relieve the pressure associated with CS has been proposed to accommodate edema after detorsion, embracing the raw fasciotomy area with tunica vaginalis flap (TVF) or graft. Fashioning the TVF can be tedious in cases of severe scrotal edema. Herein we present a technique that facilitates and expedites the procedure, maintaining the fasciotomy area decompressed.

**Materials and Methods:**

In testicular torsion, where the testis remains with dark coloration and questionable viability after detorsion a longitudinal releasing incision is made in the tunica albuginea (fasciotomy) to decrease compartmental pressure. If signs of parenchymal recovery (bleeding points, better color) are seen an orchio-septopexy is performed, suturing the incised albuginea’s edges to the septum with a running suture, avoiding CS as well as re-torsion.

**Results:**

Orchio-septopexy was performed in 11 cases with a mean age of 11.9 years (3-17). All cases had clinic follow-up and testicular Doppler US with a mean of 9.5 months (6-24). 6/11 cases (54%) were salvaged, with good vascularity in the Doppler US and maintained more than 50% testicular volume compared to the contralateral side.

**Conclusion:**

Orchio-septopexy after testicular fasciotomy is a simple and fast technique that can be utilized in cases of prolonged testicular ischemia and questionable viability. More than half of the testes recovered, encouraging us to propose its utilization as well as its validation by other surgeons.

## INTRODUCTION

Testicular torsion (TT) is a common emergency condition encountered by Pediatric urologist with annual incidence of 3.8 per 100,000 pediatric patients ( 1 ). The clinical management of TT is critical, relying on prompt assessment and surgical exploration ( 2 ). The testicle viability is time sensitive to ischemia, with rate of orchiectomy reaching 80- 90% when ischemia time exceeds 24 hours ( 3 ). Different methods were used aiming to improve the salvage rate of testis post torsion, namely: educational material for health care takers, initial care of TT cases, clinical guidelines to decrease time from emergency department door to operating room ( 4 ), manual detorsion maneuver prior to exploration ( 5 ) and incision in the testicular albuginea (TA) to relieve potential compartmental pressure and improve testicular vascularization ( 6 ). The conceptualization of testicular compartment syndrome (CS) following TT with fasciotomy to tunica albuginea to relieve that pressure has been previously validated for the management of TT with prolonged ischemia time, mobilizing a tunica vaginalis flap (TVF) to cover the fasciotomy site and to maintain lower intra testicular pressure ( 7 , 8 ). Although the construction of TVF is a relatively simple procedure, it can be time-consuming and associated with bleeding, especially if scrotal edema and inflammatory reaction is present. We designed a technique that expedites the procedure, maintaining the fasciotomy area covered and decompressed. We hypothesized that fixing the testis and attaching the fasciotomy area to the septum facilitates the technique and decompress the parenchyma, avoiding CS and improving vascularization. The objective of this report is to present a novel technique for testicular fixation in cases of torsion and to confirm usefulness of previously validated fasciotomy of tunica albuginea ( 7 - 9 ). Herein we describe the procedure and our preliminary results.

## MATERIAL AND METHODS

We performed a retrospective review of all cases with testicular torsion that underwent scrotal exploration between January 2018 and 2020. Patients with unilateral intravaginal TT and a minimum of one documented follow up visit have been included. Perinatal TT, torsion of undescended testis, TT in patients with single gonad and documented contra-lateral testicular abnormality were excluded. Patient’s demographics as well as time of evolution was noted.

### Surgical technique

**Figure 1:**
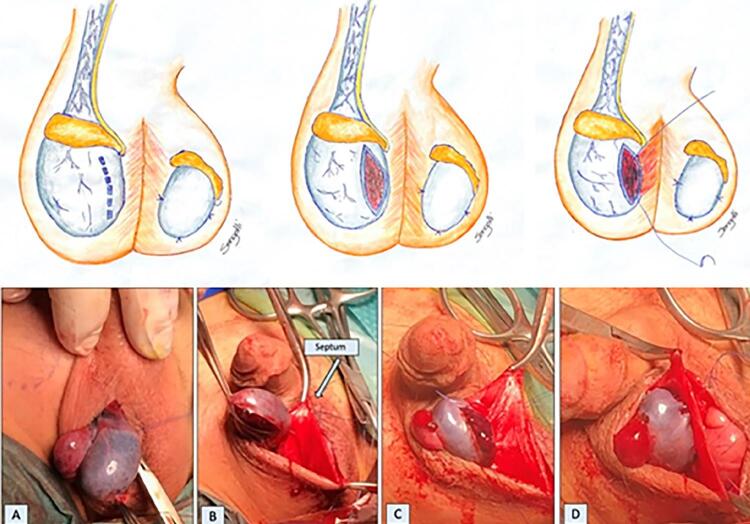
Orchio-Septopexy technique.

The scrotum is explored via midline incision, identifying and preparing the testicular septum as well as exposing the affected side first in order to confirm the diagnosis of TT. The testicle is untwisted and kept covered with gauzes soaked with warm saline. Attention is then driven to the contralateral testicle that is pexed to avoid future torsion. Then the affected side is re-assessed. If it recovers normal colour it is then pexed with monofilamentar non absorbable 5/0 sutures. If it remains darker after detorsion a generous incision in TA is made, aiming decompression and improvement of vascularization. If parenchyma remains dark and with bleeding points it is assumed that irreversible necrosis have ocurred and orchiectomy is warranted. If color improves and bleeding points appear in the parenchyma an orchio-septopexy is performed via running suture of polydioxanone 5.0 sutures , starting at the lower edges of the fasciotomy ( Figure-1 ). For additional technical details we invite the reader to view a video following the link: < https://intbrazjurol.com.br/videos/20220128_Pippi-Salle_et_al.mp4 >

A) Testis with persistent discoloration after de-torsion; B) Aspect of the testis after fasciotomy of tunica albuginea (improved coloration and with some bleeding points); C) Running suture of the lower edges of incised albuginea to midline scrotal septum; D) Orchio-septopexy completed.

**Figure 2:**
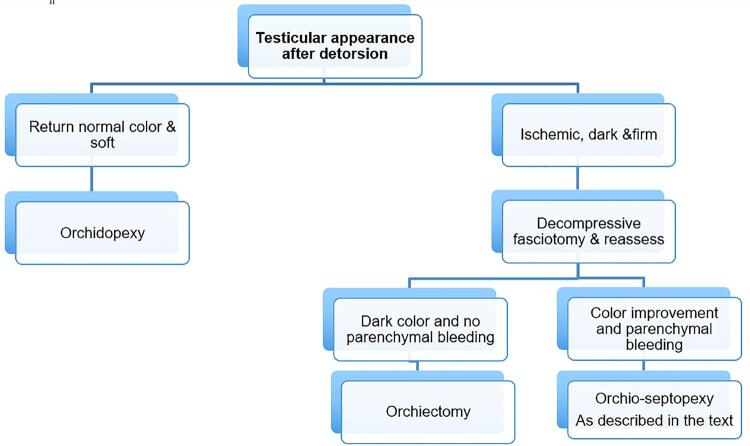
illustrates our algorithm for management of TT.

The summary of intra-operative approach of TT as shown in Figure-2 .

Summary of intra-operative approach for testicular torsion.

Post-operative physical examination as well as Doppler US were performed at least 6 months after surgery. The definition of post-operative testicular salvageability is having adequate blood flow on Doppler US, maintaining at least of 50% testicular volume compared to the contralateral side.

## RESULTS

**Figure 3:**
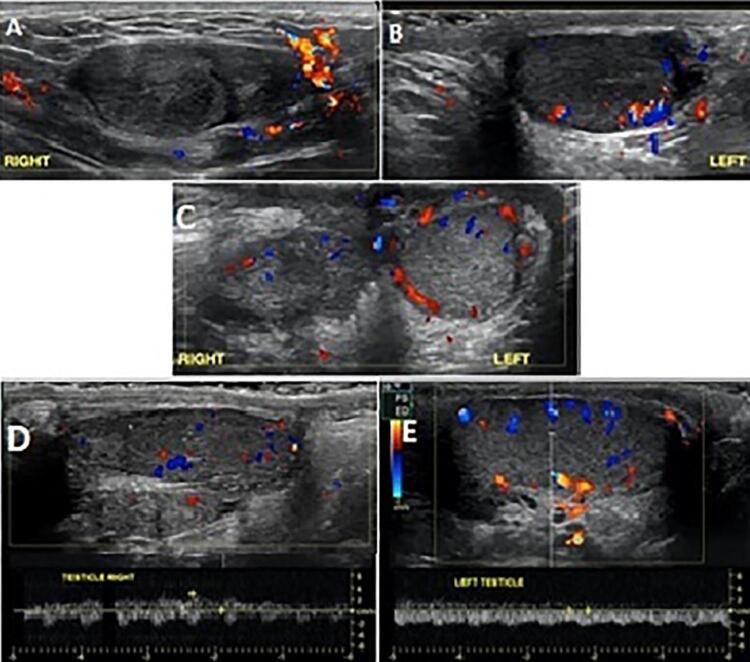
Pre and post testicular ultrasounds in a 14-year old boy with borderline viable testis after detorsion who underwent right orchio-septopexy.

Between January 2018 and 2020, 21 patients underwent scrotal exploration for TT. The age of patients ranged from 3 to 17 years old. Left sided was slightly more affected than right (12 cases, 57.14%). Five cases (23.8%) improved with detorsion alone and underwent routine bilateral orchiopexy. In 16 patients the color did not improve after detorsion and underwent fasciotomy. Dark coloration with necrotic looking parenchyma remained in 5 cases and these patients underwent orchiectomy. Eleven testicles improved the color, having points of parenchyma bleeding and underwent orchio-septopexy. All patients who underwent orchio-septopexy after fasciotomy had clinic follow-up and testicular Doppler US with a mean of 9.5 months ( 6 -24) and 6 of 11 (54%) were salvaged, having good flow, maintaining more than 50% testicular volume compared to the contralateral as illustrated in Figure-3 . Interestingly, 4 patients (66.7%) with 12 hours or less history of pain, who underwent orchio-septopexy had viable testis post operatively and also 2 (40%) with prolonged ischemia time (>12H) recovered after this procedure ( Table-1 ).

A, B) Pre-op Doppler in patient with right testicular torsion and prolonged ischemia: no flow in the parenchyma; C) Doppler 2 weeks after right orchio-septopexy showing recovery of flow; D, E) Doppler at 1 year follow-up: maintenance of good flow and more than 50 % of right testicular volume compared by left side.

**Table 1:** The correlation of the duration of pain with different surgical outcome.

Duration of pain	Bilateral Orchiopexy (Viability %)	Orchiectomy (Viability %)	Orchio-septopexy (Viability %)
6 hours or less	4	0	0
7-12 hours	1	0	6 (4 cases 66.70%)
More than 12 hours	0	5	5 (2 cases 40%)

**Total (21 cases)**	**5 (100%)**	**5(NA)**	**11 (6 cases 54.5%)**

## DISCUSSION

Compartment syndrome (CS) is a well-known and important initiator of post ischemic insult to various organs constrained by natural envelops, including but not limited to orthopedics and trauma, being well documented in the literature ( 10 ). The increase of intra compartmental pressure leads to vicious cycle of hypoxia, accumulation of lactate due to anaerobic metabolism, edema, further deterioration in the intra compartmental pressure and decrease in capillary flow ( 11 ). Similarly, the concept can be applied to testis which is constrained by the non-elastic layer of tunica albuginea. An increase in the intra-testicular blood volume due to venous congestion of the spermatic cord, in addition to tissue changes following ischemia reperfusion injury, are likely the pathophysiology of testicular CS ( 12 ). Therefore, the concept of testicular fasciotomy via incising the tunica albuginea has been introduced to break the pathological vicious cycle of testicular CS following torsion. The concept of covering the raw fasciotomy area with tunica vaginalis flap or graft (TVF) aims to maintain a low compartment pressure inside the testicle, preventing high intra testicular compartment pressure ( 9 ). However, construction of a TVF in edematous and inflamed scrotal tissue can be tedious due to blood oozing. Our technique, attaching the raw parenchymal directly to the septum instead of covering with TVF facilitates and expedites the surgery, usually done in emergency conditions, therefore useful in such conditions. This procedure was successful in 54.5% of patients with questionable viability testis after detorsion.

Although we did not measure the time spent to perform orchio-septopexy or compare with other techniques, it seems faster and easier than the TVF technique, done previously by the senior author. This modification, with a follow-up of 9.5 months, confirms the results published in previous paper from Toronto, a similar experience with 11 patients who had TVF after fasciotomy, obtaining 54% of testicular viability on Doppler US at 7.9 months mean follow-up ( 8 ).

Orchio-septopexy seems to be particularly useful in patients with prolonged clinical evolution. Our study confirms the impression of Chu, et al. who retrospectively reviewed a cohort of 182 patients who 49, 36, and 97 underwent orchiectomy, TVF and orchiopexy alone, respectively ( 13 ). In their study TVF was particularly useful in patients with prolonged ischemia but less than 24 hours evolution where 33% of testis remained viable. In our study 6 of the 11 testis treated with orchio-septopexy remained viable (54%), including 2 with more than 12h history 6 months post operatively, confirming that, recovery is possible even in severely compromised testicles treated by orchio-septopexy (Table- 1 ). It’s worth mentioning that, 81% (17 cases) of our patients had more than 6 hours history of acute scrotal pain and that explain the higher rate of fasciotomy of TA (52%) among our cases to relieve the CS associated with prolonged ischemia.

Our study has limitations: it is retrospective and reviews a small number of patients with relatively short follow-up. In addition, in order to confirm our impression that the proposed technique is faster and easier to perform, it should be ideally done in a randomized fashion with the established utilization of TVF to cover the fasciotomy area, measuring the duration of the procedures with both techniques as well as the outcomes. However, its simplicity and the encouraging early results stimulate us to propose its utilization and possible validation by other surgeons.

In conclusion, orchio-septopexy after fasciotomy of tunica albuginea is a simple and fast technique that can be utilized in cases of prolonged testicular ischemia with questionable viability. More than half of the testis recovered, encouraging us to propose it as alternative to treat testis jeopardized by compartment syndrome after detorsion.
